# TMA Vessel Segmentation Based on Color and Morphological Features: Application to Angiogenesis Research

**DOI:** 10.1155/2013/263190

**Published:** 2013-12-05

**Authors:** M. Milagro Fernández-Carrobles, Irene Tadeo, Gloria Bueno, Rosa Noguera, Oscar Déniz, Jesús Salido, Marcial García-Rojo

**Affiliations:** ^1^VISILAB, E.T.S.I. Industriales, Universidad de Castilla-La Mancha, 13071 Ciudad Real, Spain; ^2^Fundación Investigación Clínico de Valencia, Instituto de Investigación Sanitaria, INCLIVA, 46010 Valencia, Spain; ^3^Laboratorio de Patología Molecular, Departmento de Patología, Facultad de Medicina y Odontología, Universidad de Valencia, 46010 Valencia, Spain; ^4^Departmento de Anatomía Patológica, Hospital General Universitario de Ciudad Real, 13005 Ciudad Real, Spain

## Abstract

Given that angiogenesis and lymphangiogenesis are strongly related to prognosis in neoplastic and other pathologies and that many methods exist that provide different results, we aim to construct a morphometric tool allowing us to measure different aspects of the shape and size of vascular vessels in a complete and accurate way. The developed tool presented is based on vessel closing which is an essential property to properly characterize the size and the shape of vascular and lymphatic vessels. The method is fast and accurate improving existing tools for angiogenesis analysis. The tool also improves the accuracy of vascular density measurements, since the set of endothelial cells forming a vessel is considered as a single object.

## 1. Background

Angiogenesis is present during development as well as during embryogenesis and during reparative processes for wound healing. It also has a significant role during organ transplantation since new vessel formation can be crucial to successfully prevent allograft rejection. The role of angiogenesis in the pathogenesis of chronic inflammatory diseases is of considerable interest. A positive feedback has been found in which inflammatory state promotes angiogenesis and the angiogenesis in turn facilitates chronic inflammation [1, 2]. There is increasing evidence that chronic inflammation is tightly linked to diseases associated with endothelial dysfunction and plays a role in the induction of aberrant angiogenesis [3]. Lymphatic vasculature is a prerequisite for the maintenance of tissue fluid balance and immunity in the body [4].

It is now widely accepted that tumor growth and metastasis are angiogenesis and lymphangiogenesis dependent providing novel therapeutic targets in malignant disease [5–7]. A common feature of tumor vessels studies is that the investigators focus on microvessel density overlooking other parameters that might be significant, such as the size and shape of the microvessels [8]. In many aspects, tumor vessels are different from normal vessels [9, 10]. Studies have revealed the importance of the size and shape of blood vessels in, for example, laryngeal tumors [11].

To our knowledge, there are only two applications providing vessel closing when the whole perimeter of the vessels is not completely stained, which could be a basic feature in translational research. Aperio's application for angiogenesis analysis [12] is an excellent tool for managing microvessels. This software allows to perform many operations in whole slide images but the closing algorithm is not automatic. For closing vessels the user must draw manually the lost segment on the image. The second algorithm is free and available at http://www.caiman.org.uk/ [13]. The algorithm cannot process image files larger than 2 MB. Both of them measure shape parameters but are global measurements; the properties of each microvessel are not calculated individually.

Other works such as van der Laak et al. [14], Tsuji et al. [15], Laitakari et al. [11], Luukkaa et al. [16], Virgintino et al. [17], and Dagnon et al. [18] have been also developed in order to calculate morphometric measurements in microvessels. Most of them are semiautomatic and require manual interaction. Van der Laak extracts morphometric measurements like area, perimeter, convex perimeter, or circularity in microvessels but when the vessels are not closed a manual correction is performed. Selecting regions of interest and vessels is necessary in Tsuji's work. Virgintino and Dagnon divide the process into two tasks, microvessel selection and measure calculation. Virgintino applies filters and image segmentation techniques for selecting microvessels. Dagnon selects each microvessel manually and works with the grayscale image. Then, measurements of these vessels are calculated using other image analysis software, that is, VIDAS release 2.5 (Kontron Elektronik, Eching, Germany) and ImageJ software (National Institute of Health, USA). These processes do not consider open vessels.

Our aim is to develop a morphometric tool able to perform a segmentation of blood and lymphatic vessels to study vascularization following the hypothesis that tumor prognosis may not only be influenced by microvascular density but also by the shape and size of the vessels. Thus, the tool is able to deal with closed and open vessels and provide morphometric measurements for each detected vessel. Besides, the tool provides two kinds of executions, an automatic execution without user interaction and another where the user can select the vessels to be analyzed. To this end a segmentation algorithm based on two complementary methodologies has been developed to segment closed and open vessels. A description of the materials used for this work can be found in Section 2.  Section 2.1 presents the algorithm implementation and how the application works.  Section 3 shows the results for the implemented tool, called AngioPath, with a comparison of CAIMAN tool. Finally, conclusions are drawn in Section 4.

## 2. Materials and Methods 

A dataset of 700 cores extracted from 10 TMA scanned slides was considered. TMA images were formed by several vessels, from 2 to 600 vessels of different sizes and stains. The images have been stained with IHQ technique against D2-40 (lymphatic vessels) and from a previously stained TMA with anti-CD34 antibody (blood vessels). This dataset was prepared with an automatic tissue arrayer composed of 70 cores/TMA and digitized with Aperio ScanScope T2 at 40x. The resolution of Aperio ScanScope T2 at 40x objective is 0.23 *μ*m/pixel. Thus, these cores are images at 40x magnification and their size varies between 6200 and 7300 pixels.

Experiments were performed on an Intel Core i7 950 3.07 Ghz and 12 GB RAM. The method has been implemented using C/C++ and the IPP libraries for image processing. Also, the Intel TBB library has been used for parallelization of the algorithms.

### 2.1. Algorithm Implementation

Histologic sections comprise two types of vessels: vessels with unquestionable endothelial cells completely stained in their perimeter (closed vessels) and vessels whose endothelial cells do not show a completely staining reaction (open vessels). Closed vessels can present vascular lumen or not. However, open vessels must always present a vascular lumen. The database used in this study is composed of both types of vessels. In the case of closed vessels, their morphometric measurements can be easily calculated. The challenge appears when the stain is weak or the vessels are not closed. In the first case, color analysis is needed; in the second case a radial algorithm is also used.

**Figure 1:**
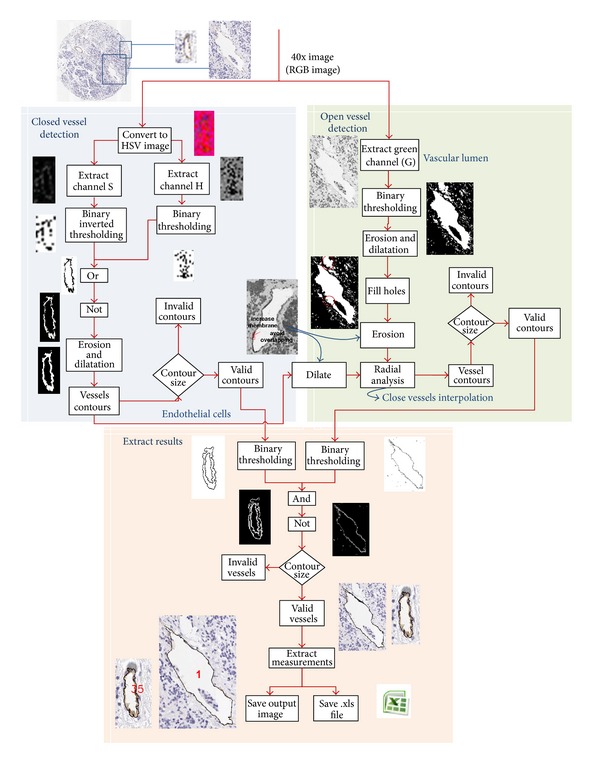
TMA blood vessel segmentation process. Division of the algorithm in two steps: first the segmentation based on HSV color model (right) and then the radial algorithm for joining open vessels.

The system developed for vessels segmentation, called AngioPath, consists of two parts: (a) color-based segmentation and (b) radial distribution of the vessel contour pixels. The algorithm is illustrated in Figure 1.

Eleven morphometric measurements are calculated for each vessel detected. The measurements describe the shape and geometrical properties of the vessels. They are briefly explained in Table 1. In addition, a summary of the measurements is also provided. The vessels are grouped by their height and the measurements are calculated for each group. There are 5 height groups: between [5 and 15 *μ*m), [15 and 20 *μ*m), [20 and 59 *μ*m), [50 and 200 *μ*m), and more than 200 *μ*m. Also, the total area, a percentage of area, and the total number of vessels are calculated for groups. Finally, an average of the eleven measurements for all vessels is calculated; thus a total of 24 morphometric measurements are provided for all the segmented vessels (see Table 2).

The methods applied for the development of AngioPath are described as follows.

#### 2.1.1. Segmentation Based on HSV Color Model

The objective of this part is brown color segmentation through HSV color model to detect the endothelial cells. Most of the closed vessels can be detected through their brown color. Open vessels undergo a radial analysis after segmentation of their endothelial cells. This algorithm proceeds as follows.

**Figure 2:**
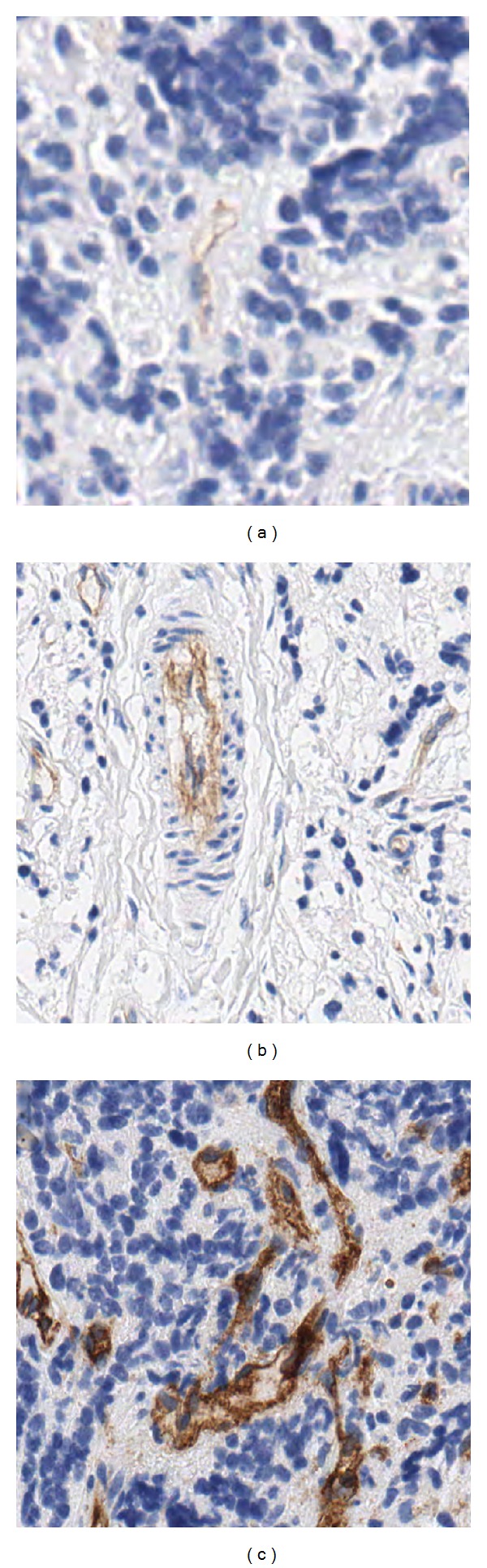
Vessels in TMA samples with different degrees of staining. (a) Weak stain, (b) normal stain, and (c) strong stain.

(1) *Conversion of the RGB TMA Image to the HSV Color Model*. This conversion is useful for segmentation of brown color; in other words, it allows extracting the vascular vessels membrane for both closed and open vessels.
(2) *Extraction of the S and H Channels from HSV Image*. The S channel contains most of the shades and brown stains, but it is not enough. Therefore, the H channel is also used.
(3) *Application of a Binary Thresholding to the H Channel Image and a Binary Inverted Thresholding to the S Channel Image*. The thresholding operation makes a comparison between the values of the image pixels and one threshold value. In case of binary thresholding (see (1)) when the value of the pixel, *I*(*x*, *y*), is larger than the established threshold value, *T*_H_, the new pixel, *I*′(*x*, *y*), will take the maximum value *M* (with *M* equal to 255). On the contrary, if the value of *I*(*x*, *y*) is lower than *T*_H_, then *I*′(*x*, *y*) will take value 0:
  (1) $$\begin{array}{c} I^{\prime }\left(x,y\right)=\{\begin{array}{cc} M & I\left(x,y\right)>T_{\text{H}},\\ 0 & \text{otherwise}. \end{array} \end{array}$$
 In the binary inverted thresholding the new pixel values, *I*′(*x*, *y*), are inverted. When the value of *I*(*x*, *y*) in the S color image is larger than the established threshold value, *T*_S_, *I*′(*x*, *y*) will be equal to 0. The pixel values lower than the threshold *T*_S_ will take value 255. Threshold values depend on the stain selected. The threshold *T*_H_ applied to the H channel is always 20 but the inverted threshold *T*_S_ applied to S channel is 10 for weak stains, 20 for normal stains, and 30 for strong stains.  Figure 2 shows samples of TMAs with different degrees of stain. Moreover, the user can select manually other values for the *T*_H_ and *T*_S_.
(4) *Application of a Logical OR Operator to Both Binary Images*. This operation segments brown color and erases the rest of the colors.
(5) *Application of a Logical NOT Operator in Order to Invert the Image*. This operation is needed to highlight the contour vessels.
(6) *Elimination of Small Artifacts of the Input Image and Joining Nearby Structures*. Erosion and dilation operations of 2 and 4 iterations, respectively, are performed in the image. Erosion, *E*(*x*, *y*) (see (2)), is done by means of a convolution where the minimum value of the neighborhood pixels are selected. The erosion allows eliminating small artifacts in the image and therefore reducing false positives. Then, the dual operation to erosion, that is, a dilation, *D*(*x*, *y*) (see (3)), is performed to join nearby structures:
  (2) $$\begin{array}{c} E\left(x,y\right)=\underset{x^{\prime },y^{\prime }\in \text{kernel}}{\min}I\left(x+x^{\prime },y+y^{\prime }\right), \end{array}$$
  (3) $$\begin{array}{c} D\left(x,y\right)=\underset{x^{\prime },y^{\prime }\in \text{kernel}}{\max}I\left(x+x^{\prime },y+y^{\prime }\right). \end{array}$$
(7) *Application of a Contour Finding Operator*. Finally, a contour finding operator is applied to find the core contours. This algorithm computes contours from binary images like images created by a Canny operator, which have edges pixel in them, or images created by a binary thresholding, in which the edges are implicit as boundaries between positive and negative regions. Then, the algorithm retrieves contours from the binary image using the algorithm of Suzuki and Abe [20]. The algorithm allows storing the vessel contour pixels through sequences and manipulating them individually.
(8) *Discarding Small Artifacts*. Contours whose length is lower than 6 pixels (1.38 *μ*m) or their width and height are higher than 20 pixels (4.6 *μ*m) are discarded. The remaining contour pixels are the valid vessels.

#### 2.1.2. Radial Distribution of the Vessel Contour Pixels

This algorithm finds the vascular lumen, which is always present in open vessels, and their brown endothelial surrounding cells. Then the unconnected parts of the vessel are joined together. Once the open vessels are closed, the morphometric measurements are calculated. The algorithm proceeds as follows.

**Figure 3:**
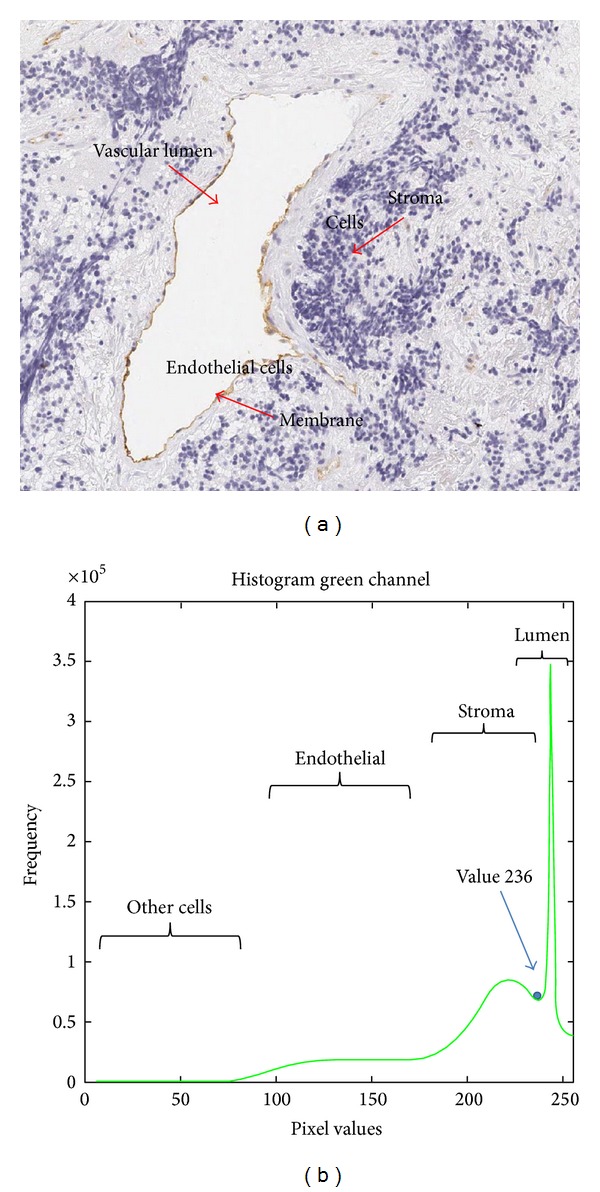
Statistical analysis of the TMA structures based on the image histogram. (a) Original TMA sample with the main structures, (b) histogram of the green channel image.

**Figure 4:**
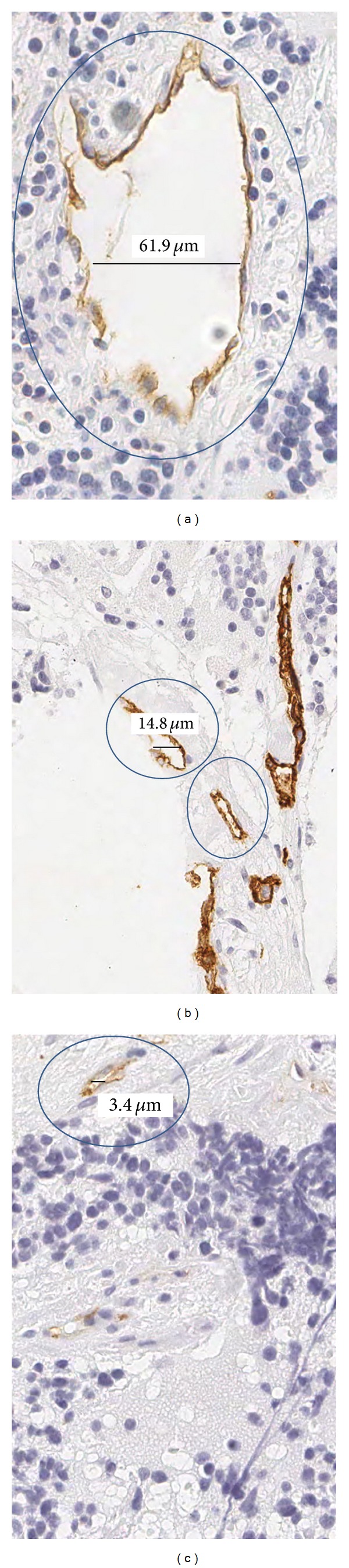
Different size of vessels to calculate the radial distribution.

1. Extraction of the green channel from RGB image, Green channel, *I*_G_, helps to distinguish the different vascular lumens at the TMA core. Besides the use of a single channel can reduce the computational time and also reduce the RAM memory used to process images.
2. A binary thresholding is applied for extracting vascular lumens at the *I*_G_ image. The threshold value was established at 236. This was done after statistical analysis of the image histogram, where it was found that the vascular lumens have a gray level larger or equal to 236; see Figure 3.
3. To join large structures and remove the smaller ones from the previous binary image, a combination of morphological transformations was applied. These transforms were erosion with 3 iterations and dilation with 2 iterations.
4. Those closed contours that have internal holes smaller than a minimum size of about 92 *μ*m (400 pixels) are filled.
5. Again, an erosion of 1 iteration is performed in the image. This erosion allows creating space between the vascular lumen and the vessel membrane.
6. Radial analysis consists of computing the normal direction for each vascular lumen point on the border with a possible vessel point. Then, the radial direction is used to check if there is any part of the membrane vessel nearby within a radio of 3 *μ*m. To this end the endothelial cells detected in the previous algorithm are also used. Notice that a dilatation of 2 iterations is performed to the endothelial cells to avoid overlapping with the vascular lumen.
7. A vessel is considered valid depending on the ratio of checked pixels that actually belong to the membrane vessel. The ratio is adjusted depending on the length of the vascular lumen contour, being equal to 60%, 50%, and 40% for small, medium, and big vessels, respectively. Small vessels are those with a vascular lumen contour length lower than 12.42 *μ*m, medium vessels between 12.42 *μ*m and 31.05 *μ*m, and large vessels greater than 31.05 *μ*m.  Figure 4 shows different vessels classified by size. Once a pixel is considered as a valid vessel a linear interpolation is done to close the open vessels.
8. Contours finding is a transformation is applied to find the vessel contours obtained in the radial analysis. This procedure is similar to point (7) of the color-based segmentation algorithm.
9. The small artifacts are removed. This procedure is similar to point (8) of the color-based segmentation algorithm.

#### 2.1.3. Extraction of Results

At this point, the algorithm provides two images composed of closed and open vessels. In the following steps both images are joined together and the morphometric measurements are calculated.

(9) A binary thresholding is applied on the previous two images. This thresholding is performed in order to obtain a binary image with only the vessel contours; therefore the threshold value is 0.
(10) Application of a logical AND operator to both images combines into the same image those vessels segmented by the HSV color model and those obtained by the radial distribution analysis.
(11) Application of a logical NOT operator is done in order to invert the image and highlight the contour vessels.
(12) A contour finding algorithm is applied to find the vessel contours of the final image. This procedure is similar to point (7) of the color-based segmentation algorithm and the redial analysis.
(13) For each valid vessel, its position by means of its center pixel and twelve morphometric measurements are given. Morphometric measurements are also provided for each group of vessels according to their height.
(14) The algorithm provides 2 outputs: (a) all morphometric measurements that are saved in an Excel format file and (b) the final image with the vessel segmented and labeled. The final image is stored in a tiff format file.

### 2.2. How Does the Application Work?

**Figure 5:**
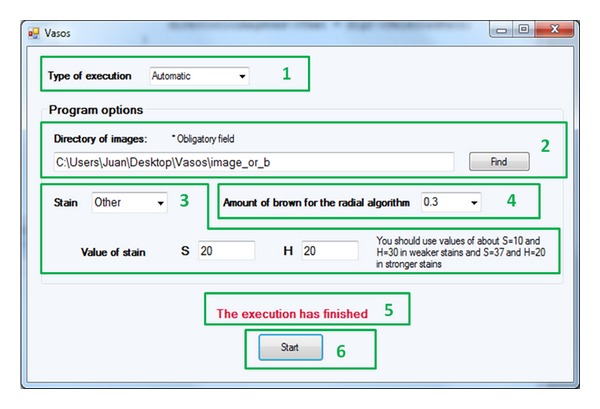
AngioPath graphical user interface. Interface parameters that can be selected or modified by the user.

AngioPath has an intuitive interface through which the user selects the execution parameters (see Figure 5). These parameters are the following.

**Figure 6:**
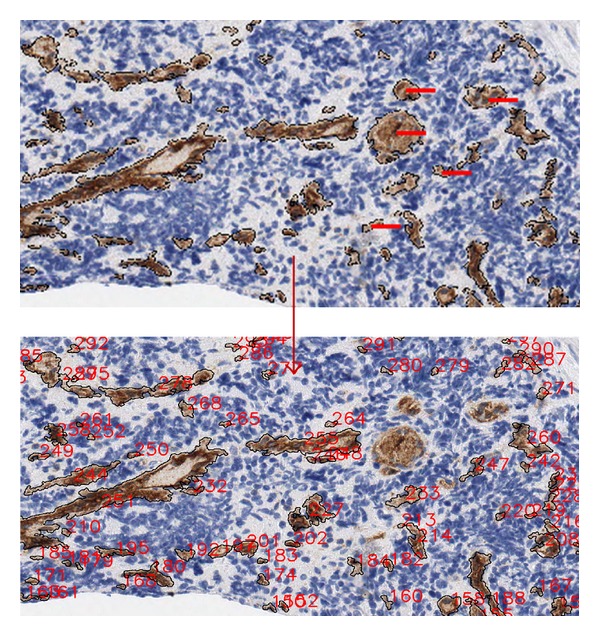
Working session with AngioPath in manual mode. Selection of nondesired vessels to be eliminated in the TMA image.

1. *Type of Execution: Automatic or Manual*. The system provides automatically an output with all vessels and their morphometric measurements. However, in order to reduce false positives which may occur in the radial analysis, the algorithm allows a manual mode. In manual mode, the user selects the nondesired vessels just after the AND operation, see Figure 1. During the execution, a window with the vessels found is displayed. At that time, the nondesired vessels are selected. This selection is made with mouse clicks on the center point of the vessel and immediately the selected vessel is marked. When all nondesired vessels have been selected the user must press the enter key or close the window and the selected vessels will be removed.  Figure 6 illustrates the manual mode with a TMA sample where 5 vessels have been selected as nondesired.
2. *Directory of Images*. It is compulsory to indicate the directory where the images are stored. To this end, the *Find* option allows browsing through the file system. The path selected is displayed in the corresponding text box.
3. *Type of Stain*. The user can make use of default values for the H and S thresholds or rather define them. Default values depend on three different stain options for weak (WS), medium (MS), and strong (SS) stain. The threshold values can also be selected by the user with *Other* option. The interface shows the variables S and H to enter their value when *Other* option is selected. Then, the new threshold values, *T*_S_ and *T*_H_, can be entered.
4. *Percentage of Stained Vessels*. This option is a list of percentages between 0.1 and 0.9. The user can specify the minimum and maximum amount of endothelial cells needed to close the vessels. Thus, the minimum amount of brown pixels is 0.1 and the maximum amount is 0.9. This percentage is related to the intensity of the stain (see Figure 2).

**Figure 7:**
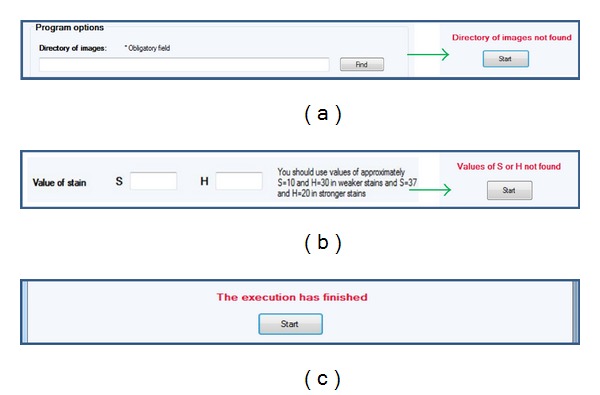
Warning labels. (a) *Directory of images not found* is displayed when the *Start* option is selected and the directory of images has not been previously selected. (b) *Values of S or H not found* is displayed when the *Other* option of stain is selected but the threshold values for S or H have not been set and (c) *the execution has finished* is displayed when all the images have been processed.

The system also provides warning labels. There are three types of warnings: (a) if the *Start* option is selected and the directory of images has not been previously selected, (b) if the *Other* option of stain is selected but the threshold values for S or H have not been set, and (c) if the process has finished, that is all vessels for all TMA images have been detected and measured.  Figure 7 shows the warning labels.

**Figure 8:**
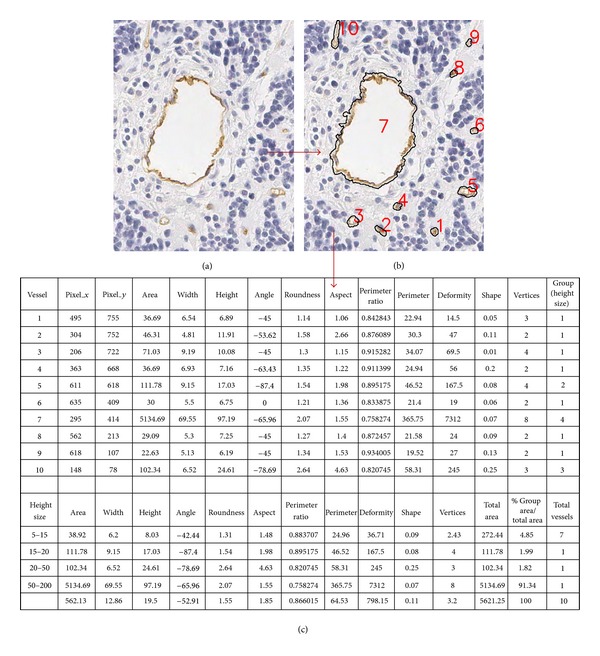
Results provided by AngioPath. (a) Original image. TMA sample with 10 vessels, (b) segmented vessels and labeled and (c) morphometric measurements for each vessel and for each group of vessels according to their height.

When all the options have been set, the *Start* button initiates the execution. An example of the final result obtained with AngioPath is illustrated in Figure 8. Figure 8 shows a TMA sample with 10 vessels and their morphometric measurements.

## 3. Results 

AngioPath has been compared with the free available algorithm CAIMAN in terms of speed, accuracy, morphometric measurements, and other properties such as the maximum allowed image size and the modes of execution. A summary of the results of this comparison is shown in Table 3. The comparison was done with a subset of 40 TMA subsamples composed of 23 samples from our database and 17 from the CAIMAN database.

Our method takes between 10 and 180 seconds for images with 2 and 600 vessels, respectively. The algorithm accepts images of any size. The average image size of our dataset is 6300 × 6300 pixels, that is, 120 MB. CAIMAN takes an average of 230 seconds for a 1.5 MB image, that is, about 1200 × 960 pixels. CAIMAN could not handle the image size of our dataset since the maximum size allowed is 2 MB.

Among the parameters measured, the shape factors roundness and aspect are calculated in both algorithms. Moreover, AngioPath includes perimeter-ratio which represents the regularity of the contour of the vessels and which we found to be related to clinical-biological features in, at least, neuroblastic tumors. AngioPath provides in total 24 morphometric measurements and CAIMAN provides 5 morphometric measurements and 2 related to density, that is, the average stained area (lumen excluded) and the average vessel area (lumen included).

**Figure 9:**
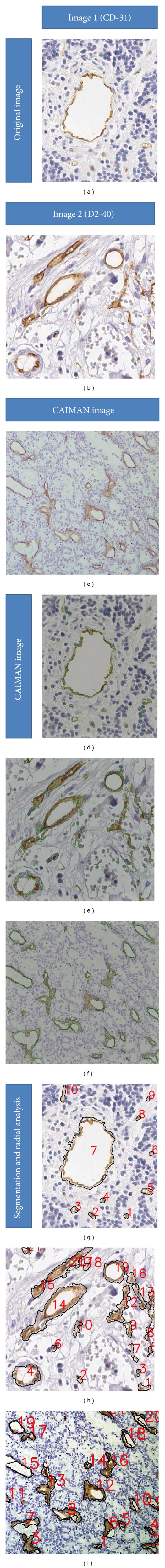
CAIMAN application. (1st row) original images: (a) and (b) are images from our database and (c) is an example image provided by CAIMAN with a less-lighted caption. (2nd row) segmentation performed by CAIMAN (green line) which does not detect small or too large vessels (d) and sometimes divide large vessels in small ones or segment blue areas as vessels (e). Their image is well segmented (f). (3rd row) results provided by AngioPath with the color-based segmentation and the radial analysis.

Our algorithm runs in manual mode which allows to correlating a measurement with a given vessel and eliminating it. This solves the problem of false positives when segmenting nondesired vessels. Both CAIMAN and the tool developed herein show a similar percentage of contour pixels correctly detected in automatic mode, 95.92% and 96.82%, respectively. However, CAIMAN has a larger number of false positives; the specificity of CAIMAN is 80% against 98.75% of AngioPath. CAIMAN does not detect small or too large vessels and sometimes takes blue areas as vessels. An example of the results obtained with both systems is shown in Figure 9 with 3 TMA subsamples, 2 from our database and 1 from CAIMAN database. CAIMAN provided better results when segmenting images from their own database. The discrepancies could probably be related to a specific and differently designed brown color spectrum given by the stain or the digital image quality. Nevertheless, the morphometric tool described herein has shown better results in speed and accuracy. An average value of 97.78% accuracy was obtained for AngioPath against 87.96% for CAIMAN.

Finally, AngioPath has been validated with 700 cores obtained from whole slide TMA images [21] by means of a ROC analysis. An average of 97% sensitivity and 99% specificity was obtained.

## 4. Conclusions 

This paper has described a morphometric tool implemented to measure different aspects of the shape and size of vascular vessels in a complete and accurate way. The developed tool takes into account both closed and open vessels. Vessel closing is an essential property to properly quantify and characterize the shape and size of vascular and lymphatic vessels. In the same way, the set of endothelial cells forming a vessel are considered together as a single object, making vascular density measurement more accurate. The tool, called AngioPath, is able to detect vessels in whole slide TMA images with an average accuracy of 97.89%. Moreover, AngioPath provides 24 morphometric measurements of the detected vessels.

Although AngioPath has shown encouraging results in the database tested, it may be improved by applying invariant color analysis techniques to properly segment vessels with different stain. By applying this tool it is expected that further studies can be carried out to test whether shape and size measurements are as important for prognosis as literature suggests.

## Figures and Tables

**Table 1:** Morphometric measurements.

Measurements	Meaning	Units
Localization	*x*-*y* coordinates	Pixels
Area	Vessel contour area	Physical units (*μ*m^2^)
Size	Vessel width and height	Physical units (*μ*m)
Perimeter	Perimeter	Physical units (*μ*m)
Angle	Angle between the horizontal axis and the first side (i.e., length)	Radians
Vascular density	$\frac{\text{Number of vessels}}{\text{core}}$	Physical units (vessel number/*μ*m^2^)
Aspect	${\frac{\text{Major}\;\text{axis}}{\text{minor}\;\text{axis}}}^{*}$	
Roundness	$\frac{\text{Perimete}{\text{r}}^{2}}{4*\pi *\text{area}}$	
Perimeter ratio	$\frac{\text{Convex}\;\text{perimeter}}{\text{perimeter}}$	
Deformity	Convex area − area	Physical units (*μ*m)
Shape	$\underset{i=1,2}{\max}\frac{\left|m_{i}^{A}-m_{i}^{B}\right|}{\left|m_{i}^{A}\right|}$	
Vertices	Calculates the approximate contour polygon vessel with less distance between vertices using the Douglas-Peucker algorithm [19]	

*m*_*i*_^*A*^ = sign⁡ (*h*_*i*_^*A*^) · log⁡ (*h*_*i*_^*A*^) and *m*_*i*_^*B*^ = sign⁡ (*h*_*i*_^*B*^) · log⁡ (*h*_*i*_^*B*^)*h*_*i*_^*A*^.

*h*_*i*_^*B*^ are the Hu moments of the normal and convex contour area (*A*, *B*), respectively, and *i* represents the seven Hu invariant moments.

*These axes correspond to the best-fitting ellipsoid of the vessel contour.

**Table 2:** Summary of measurements.

Height (*μ*m)	Ratio measurements	Total	Percentage	Vessels
[5–15), [15–20), [20–50), [50–200), ≥200	$\frac{\sum ‍\text{area}\;\text{of}\;\text{grou}{\text{p}}_{i}}{\text{total}\;\text{vessels}\;\;\text{of}\;\text{grou}{\text{p}}_{i}}$, $\frac{\sum ‍\text{width}\;\text{of}\;\text{this}\;\text{group}}{\text{total}\;\text{vessels}\;\text{of}\;\text{this}\;\text{group}}$, $\frac{\sum ‍\text{height}\;\text{of}\;\text{grou}{\text{p}}_{i}}{\text{total}\;\text{vessels}\;\text{of}\;\text{grou}{\text{p}}_{i}}$, $\frac{\sum ‍\text{angle}\;\text{of}\;\text{grou}{\text{p}}_{i}}{\text{total}\;\text{vessels}\;\text{of}\;\text{grou}{\text{p}}_{1}}$, $\frac{\sum ‍\text{roundness}\;\text{of}\;\text{grou}{\text{p}}_{i}}{\text{total}\;\text{vessels}\;\text{of}\;\text{grou}{\text{p}}_{i}},\ldots$	Total feature_*j*_ in group_*i*_	$\frac{\sum ‍\text{area}\;\text{of}\;\text{grou}{\text{p}}_{i}}{\text{total}\;\text{area}}*100$	Total vessels group_*i*_
	(*i* = 1,…, 5)		(*j* = 1,…, 12)	

Average	$\frac{\sum ‍\text{areas}}{\text{total vessels}}$, $\frac{\sum ‍\text{width}}{\text{total vessels}}$, $\frac{\sum ‍\text{height}}{\text{total vessels}}$, $\frac{\sum ‍\text{featur}{\text{e}}_{j}}{\text{total vessels}},\ldots$	Total area	Total percentage of areas	Total vessels

**Table 3:** Comparison of AngioPath versus CAIMAN.

	AngioPath	CAIMAN
Speed	0.27 s/MB	124 s/MB
Measurements	24 features	7 features
Mode	Automatic & Manual	Automatic
Maximum size	No limit	2 MB
Sensitivity	96.82%	95.92%
Specificity	98.75%	80.00%
Accuracy	97.78%	87.96%
